# Phloem Exit as a Possible Control Point in Selective Systemic Transport of RNA

**DOI:** 10.3389/fpls.2021.739369

**Published:** 2021-11-26

**Authors:** Alexander A. Lezzhov, Sergey Y. Morozov, Andrey G. Solovyev

**Affiliations:** ^1^Faculty of Bioengineering and Bioinformatics, Moscow State University, Moscow, Russia; ^2^A.N. Belozersky Institute of Physico-Chemical Biology, Moscow State University, Moscow, Russia; ^3^Department of Virology, Faculty of Biology, Moscow State University, Moscow, Russia

**Keywords:** phloem, phloem transport, mobile RNA, systemic signaling, phloem exit, plasmodesmata, phloem transport signal

Phloem sieve tubes constitute the route for the transport of photosynthesis products and consist of sieve elements (SEs), highly specialized cells that degrade the nucleus and some other organelles when they differentiate (Lucas et al., 2013). Therefore, the maintenance of mature SEs is assumed to depend on the transport of essential proteins from companion cells (CCs) connected to SEs by specialized pore plasmodesmata with a high size exclusion limit of 70 kDa (Oparka and Turgeon, 1999). As suggested by analyses of phloem exudate preparations, enucleate mature SEs incapable of transcription contain a plethora of RNA (Kehr and Kragler, 2018; Maizel et al., 2020), raising questions on the origin and functions of RNA in the phloem sieve tubes that are still largely unanswered. Here, we discuss the concept of selective RNA transport through the phloem directed by specific signals in RNA molecules.

RNA in the phloem translocation stream is assumed to be transported to distant plant parts. For a number of RNA species, including both mRNAs and small RNAs (Lin et al., 2008; Pant et al., 2008; Hannapel and Banerjee, 2017), long-distance transport has been documented to take part in plant development and responses to external stimuli (Lough and Lucas, 2006; Lucas et al., 2013; Maizel et al., 2020).

In some mRNAs, regions responsible for their ability for transport through the phloem or “phloem transport signals” (PTSs) have been mapped. One known PTS type is represented by polypyrimidine tracts found in mRNAs of pumpkin CmGAI and CmPP16-1 proteins (Xoconostle-Cázares et al., 1999; Ham et al., 2009) and potato StBEL5 and POTH1 proteins (Banerjee et al., 2006; Mahajan et al., 2012). In pumpkin, polypyrimidine tracts in phloem-mobile RNAs can be bound by RBP50, a phloem polypyrimidine-binding protein, serving as a core component in a multiprotein ribonucleoprotein (RNP) complex, presumably the RNA transport form (Ham et al., 2009). Another PTS type is represented by diverse highly-structured RNA regions such as tRNA sequences in untranslated regions of certain mRNAs, tRNA-like structures in RNA genomes of particular plant viruses, micro-RNA precursors, and viroids (Tolstyko et al., 2020). Although not studied in detail, these structures are shown to mediate phloem transport of native RNAs (Gopinath and Kao, 2007; Ding, 2009; Zhang W. et al., 2016) and heterologous reporter RNAs (Zhang W. et al., 2016; Lezzhov et al., 2019).

The model of specific phloem transport of PTS-containing RNAs does not fully comply with the available data. Phloem exudate preparations contain thousands of mRNA species, as well as rRNA, tRNA, snRNA, and other non-coding RNAs in their native and truncated forms (Zhang et al., 2009; Kehr and Kragler, 2018). These RNAs can originate from CCs and transcription-competent immature SEs, which release RNA into the phloem translocation stream upon their maturation and simultaneous connection to the conducting phloem (Knoblauch et al., 2018). Grafting experiments identify hundreds to thousands of mRNAs transported between rootstocks and scions (Kehr and Kragler, 2018). Although the RNA mobility does not always correlate with the presence of PTS (Xia et al., 2018), it correlates, according to computer modeling, with RNA stability and abundance in CCs (Calderwood et al., 2016). However, high-level expression of heterologous RNAs in CCs cannot ensure their phloem transport (Paultre et al., 2016; Xia et al., 2018), suggesting that the mechanistic abundance-dependent model requires verification. In any case, the concept of PTS-dependent transport, being valid to describe long-distance translocation of certain PTS-containing RNAs, fails to explain the abundant RNA presence in the SEs and massive RNA transport through the phloem.

Exact PTS functions are unknown. A possible PTS role in the RNA translocation from CCs to SEs can be envisaged; however, experimental data supporting this hypothesis are lacking. As the repertoire of phloem RNAs and mobile RNAs is changed in response to external stimuli, such as Pi or nitrogen starvation and draught (Thieme et al., 2015; Zhang Z. et al., 2016; Hao et al., 2020), a hypothesis of conditional mRNAs mobilization for the long-distance transport has been put forward, suggesting that immobile PTS-containing RNAs can acquire phloem mobility under certain conditions (Thieme et al., 2015; Zhang Z. et al., 2016; Xia et al., 2018), possibly when the expression of PTS-binding proteins enabling RNA entry into SEs is upregulated in CCs. In addition, PTSs may interact with RNA-binding proteins, which increase RNA stability in the phloem translocation stream and thus facilitate RNA phloem transport. Although RNases are not found in phloem exudates (Kehr and Kragler, 2018), mobile mRNAs are shown to undergo degradation during their movement in grafted plants (Xia et al., 2018). Accordingly, polypyrimidine-binding proteins StPTB1 and StPTB6 bind to potato BEL5 RNA and increase its stability during phloem transport to stolons (Cho et al., 2015).

Other PTS roles can be suggested based on studies of viroids, which are circular highly-structured infectious non-coding RNAs capable of replication and transport in plants (Ding, 2010). Potato spindle tuber viroid (PSTVd) RNA contains a complex signal essential for PSTVd translocation from phloem bundle sheath cells to CCs and SEs (Zhong et al., 2007; Wu et al., 2020). Importantly, a specific signal in PSTVd RNA enables its exit from vascular bundle after delivery to distant plant parts, particularly, the exit from bundle sheath cells to mesophyll cells (Qi et al., 2004). Thus, the exit to destination tissue is a controlled process that requires an exit signal in the phloem-mobile RNA, likely interacting with protein factor(s) enabling translocation over boundaries between particular cell types. This hypothesis is supported by studies of *Citrus exocortis viroid* (CEVd). In the leaves of systemically infected *Citrus medica* plants, CEVd is able to exit from vascular bundles and infect mesophyll cells, whereas in *Citrus karna*, CEVd is restricted to vascular tissues. In grafting experiments, when a non-infected *C. medica* scion has been grafted onto a CEVd-infected *C. karna* rootstock, new leaves of the scion, as expected, become infected with the viroid. More important, CEVd in the rootstock acquires the ability to exit from vascular bundles to the mesophyll (Bani-Hashemian et al., 2015). These data show that a phloem-mobile factor enabling exit to mesophyll can be transported from the scion to the rootstock. We hypothesize that such factor can be a protein that specifically binds to a CEVd phloem exit signal, which is conceivably similar in functions to that in PSTVd, and directs translocation of CEVd RNA out of the vascular bundle. It seems probable that PTSs in many phloem-mobile mRNAs can have similar functions, serving as elements enabling selective exit from the phloem.

In apical parts of growing roots and shoots, phloem unloading occurs through protophloem SEs (PSE), which serve as a transit point between the conducting phloem and surrounding sink tissues (Oparka et al., 1994). Recent detailed studies of phloem unloading in *Arabidopsis* root tips have revealed that proteins are unloaded from PSE into phloem pole pericycle (PPP) cells, rather than into CCs, through unique funnel plasmodesmata (Ross-Elliott et al., 2017). Further, small proteins freely diffuse through ordinary plasmodesmata connecting PPP with surrounding cells, whereas larger proteins with MW ≥ 48 kDa are retained in the PPP cells (Ross-Elliott et al., 2017). Although the phloem transport of RNA has not been analyzed in these experiments, mobile RNAs likely follow the route of proteins, since RNAs are believed to be transported through the phloem in the form of RNP complexes (Lough and Lucas, 2006). Due to their big size, exit of RNAs or transport RNP complexes from root tip PPP cells can unlikely occur by diffusion and should be an active process involving proteins that would selectively bind PTS signals in RNAs destined for exit into recipient tissues and mediate their transport out of PPP cells. Therefore, a PTS-based selection of RNA molecules can take place after RNA exit from phloem conductive elements. Details on the route of phloem unloading are known for *Arabidopsis* root tip only. However, we hypothesize that a similar selection mechanism can operate in other organs and tissues. In leaf minor veins, for example, a PTS-based RNA selection can occur in bundle sheath cell, the point where the transport of PSTVd out of vascular bundle can be blocked by mutations in PSTVd RNA (Qi et al., 2004).

Therefore, we propose the following hypothetic model of PTS-directed selective RNA transport in the phloem. We assume that RNA in the SEs is a complex mixture of molecules (Figure 1), consisting of minor amounts of PTS-containing RNA destined for delivery to distant plant organs and bulk of RNA that have entered the SEs non-specifically, likely from both CCs or maturing SEs, and reside in the phloem translocation stream without serving any particular function (Morris, 2018). The latter RNAs are described as superfluous RNAs (Lee and Frank, 2018). During the transport in the phloem translocation stream, RNAs targeted to distant plant organs have better chances to reach the destination point, as they may be bound by PTS-interacting proteins, such as polypyrimidine-binding proteins, which protect these RNAs from degradation, whereas unprotected superfluous RNAs may degrade during the transport (Figure 1). Upon exit from the phloem to a “sorting point” (Figure 1), for example, to the PPP cells of root tips, RNAs destined for delivery to target tissues may be selected for transport out of vascular bundle due to the presence of PTS enabling crossing the respective cell boundary, while superfluous RNAs degrade without exit to surrounding tissues, likely serving as an important source of ribonucleotides (Lee and Frank, 2018). Thus, according to this model, the PTS-based RNA selection for phloem egress determines the fate of RNA delivered through the phloem to distant tissues, and therefore exit from the phloem can be considered as one of the key control points in the phloem RNA transport. Conceivably, different plant organs contain non-identical sets of PTS-binding proteins involved in selection of RNA molecules for exit to surrounding tissues that would explain the fact that distinct subsets of mobile mRNAs are delivered to different target organs (Thieme et al., 2015).

**FIGURE 1:**
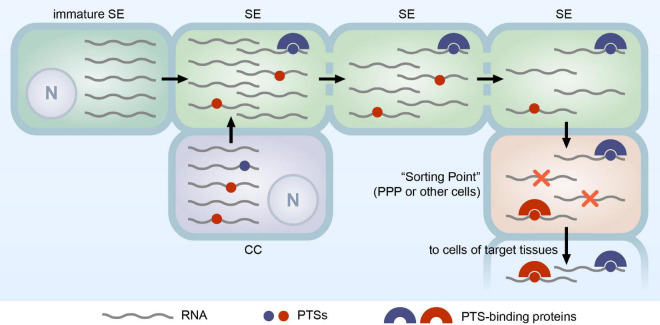
Hypothetical model of PTS-mediated selective RNA transport through the phloem. The RNA population in mature SEs consists of molecules synthesized in immature SE and CC. Some RNA species in this population contain PTSs (shown as red and blue circles). The PTS-containing RNAs may be transferred from CCs and/or immature SEs with the bulk of RNA not destined for delivery to distant plant organs. As an alternative, a selective transport of PTS-containing RNAs from CCs into SEs has been suggested, although so far not supported by experimental data. Once in the sieve tubes, RNA can be transported to distant plant organs. Many RNAs may degrade during the transport (shown as a decrease in the number of RNA molecules *per SE*). During transport, RNAs containing PTSs (blue circles) can be stabilized by interaction with PTS-binding proteins (blue half-rings) resulting in formation of transport complexes. In target tissues, RNAs are transported from the SEs into a “Sorting Point,” cells where the further fate of the delivered RNAs is determined. In the case of root tips, the “Sorting Point” is PPP cells, whereas in other organs, such as leaf minor veins, cells of other types may play this role. In the “Sorting Point,” PTS-binding proteins may enable the selective exit of PTS-containing RNAs into surrounding cells. RNAs lacking PTSs are supposed to be confined in the “Sorting Point” and degrade (shown by red crosses). In this model, PTS-binding proteins enabling RNA delivery to destination tissues have two functional roles, (i) protection of RNA during the translocation through the phloem and (ii) the selective cell-to-cell transport of PTS-containing RNAs from the “Sorting Point” to cells of a target tissue; however, it is not clear whether both roles can be played by a single PTS-binding protein. N, nucleus.

It remains unclear whether the proposed model can be relevant to both types of long-distance RNA transport described for plants, namely, the transport with and against the source-to-sink phloem flow (Kehr and Kragler, 2018). The mechanism of the latter type of transport is unknown, and some authors have hypothesized that it occurs *via* cell-to-cell movement involving non-SE cells, rather than *via* the sieve tubes (Kondhare et al., 2021). However, plant proteins, which would enable specific long-distance cell-to-cell transport of numerous dissimilar RNAs capable of sink-to-source transport, are not known. Additionally, the cell-to-cell movement-based long-distance RNA transport is very slow: the genomic RNA of tobacco mosaic virus mutants deficient in phloem transport (but capable of cell-to-cell transport due to virus-encoded movement protein) are delivered from infected lower plant leaves to upper leaves for as long as 9–12 weeks (Culver and Dawson, 1989). Therefore, we assume that the hypothesized cell-to-cell movement mechanism of sink-to-source RNA transport seems unlikely. Alternatively, RNAs that have entered the SEs can be transported along the sieve tubes in the sink-to-source direction by use of the mechanism, which is described for intracellular transport of RNA and involves the endoplasmic reticulum (ER):actin network (Pitzalis and Heinlein, 2017). Indeed, SEs contain the parietal ER, which retains continuity with ER tubules of CCs (Lucas et al., 2013) and actin microfilaments (Hafke et al., 2013). We hypothesize that the sink-to-source RNA transport occurs within sieve tubes along the ER:actin network as a specific active process that may involve so-far unidentified proteins mediating RNA translocation, and such mechanism is possible for the transport in source-to-sink direction as well. This idea agrees well with the observation that a single set of mobile RNAs is capable of both source-to-sink and sink-to-source translocation (Thieme et al., 2015; Xia et al., 2018), suggesting a functional equivalence of these types of transport. Therefore, if the RNA systemic transport in both directions occurs within sieve tubes, the proposed role of selective PTS-dependent exit from the phloem can be universal for the long-distance RNA transport in plants.

In conclusion, the functions of PTSs in the transport of phloem-mobile RNAs remain to be experimentally determined, and the proposed model may provide a useful conceptual framework for future studies.

## Author Contributions

All authors wrote the manuscript and approved it for publication.

## Conflict of Interest

The authors declare that the research was conducted in the absence of any commercial or financial relationships that could be construed as a potential conflict of interest.

## Publisher’s Note

All claims expressed in this article are solely those of the authors and do not necessarily represent those of their affiliated organizations, or those of the publisher, the editors and the reviewers. Any product that may be evaluated in this article, or claim that may be made by its manufacturer, is not guaranteed or endorsed by the publisher.
